# Interlaboratory evaluation of a digital holographic microscopy–based assay for label-free in vitro cytotoxicity testing of polymeric nanocarriers

**DOI:** 10.1007/s13346-022-01207-5

**Published:** 2022-07-08

**Authors:** Anne Marzi, Kai Moritz Eder, Álvaro Barroso, Ane Marit Wågbø, Ýrr Mørch, Anne Rein Hatletveit, Torkild Visnes, Ruth B. Schmid, Geir Klinkenberg, Björn Kemper, Jürgen Schnekenburger

**Affiliations:** 1 Biomedical Technology Center (BMTZ) of the Medical Faculty, University of Muenster, 48149 Muenster, Germany; 2 Department of Biotechnology and Nanomedicine, SINTEF Industry (SINTEF), 7034 Trondheim, Norway

**Keywords:** Digital holographic microscopy, Quantitative phase imaging, Label-free cytotoxicity testing, Nanoparticles, Interlaboratory comparison, In vitro, Regulatory science, Technology transfer

## Abstract

State-of-the-art in vitro test systems for nanomaterial toxicity assessment are based on dyes and several staining steps which can be affected by nanomaterial interference. Digital holographic microscopy (DHM), an interferometry-based variant of quantitative phase imaging (QPI), facilitates reliable proliferation quantification of native cell populations and the extraction of morphological features in a fast and label- and interference-free manner by biophysical parameters. DHM therefore has been identified as versatile tool for cytotoxicity testing in biomedical nanotechnology. In a comparative study performed at two collaborating laboratories, we investigated the interlaboratory variability and performance of DHM in nanomaterial toxicity testing, utilizing complementary standard operating procedures (SOPs). Two identical custom-built off-axis DHM systems, developed for usage in biomedical laboratories, equipped with stage-top incubation chambers were applied at different locations in Europe. Temporal dry mass development, 12-h dry mass increments and morphology changes of A549 human lung epithelial cell populations upon incubation with two variants of poly(alkyl cyanoacrylate) (PACA) nanoparticles were observed in comparison to digitonin and cell culture medium controls. Digitonin as cytotoxicity control, as well as empty and cabazitaxel-loaded PACA nanocarriers, similarly impacted 12-h dry mass development and increments as well as morphology of A549 cells at both participating laboratories. The obtained DHM data reflected the cytotoxic potential of the tested nanomaterials and are in agreement with corresponding literature on biophysical and chemical assays. Our results confirm DHM as label-free cytotoxicity assay for polymeric nanocarriers as well as the repeatability and reproducibility of the technology. In summary, the evaluated DHM assay could be efficiently implemented at different locations and facilitates interlaboratory in vitro toxicity testing of nanoparticles with prospects for application in regulatory science.

**Graphical abstract:**

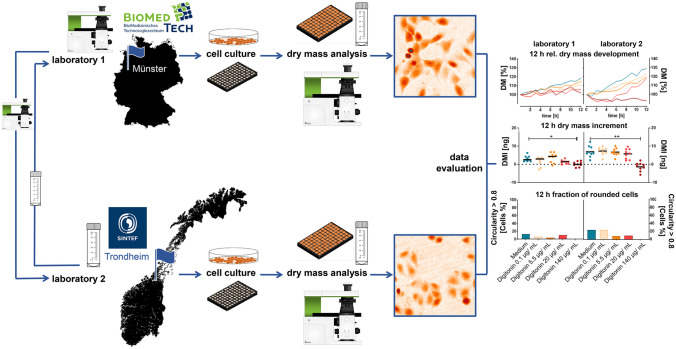

**Supplementary Information:**

The online version contains supplementary material available at 10.1007/s13346-022-01207-5.

## Introduction

The field of nanotechnology research in biomedical and pharmaceutical sciences is growing rapidly, and the number of approved nanobased pharmaceutical products increases continuously. Within the recent decade, more than 100 nanomedical applications and products have been approved for commercialization by the U.S. Food and Drug Administration (FDA) [1]. Nanomedicines can be applied as systems for gene therapy [2, 3] or drug delivery [4–6] or, for example, can serve as imaging contrast agents [7–9]. Due to their physico-chemical properties nanomaterials provide various benefits, e.g., the potential to interact with cells, organs and molecules and the ability to overcome natural barriers such as the blood–brain barrier [10, 11]. Most common nanopharmaceuticals are nanocrystals, liposomes and lipid nanoparticles, polyethylene glycol–modified polymeric nanopharmaceuticals and protein-based and metallic nanoparticles [12]. Recently, the prominent role of medical nanotechnology in current science and medicine was highlighted by the lipid nanoencapsulation of the mRNA-based COVID 19 vaccine [13, 14]. The characterization of nanomaterial effectiveness and safety are essential issues in pharmaceutical research during the development of new agents to achieve minimized side effects and maximized clinical benefit [12, 15]. Therefore, with increasing utilization of nanoparticles for medical purposes, and the minimization of possible adverse effects, extensive preclinical testing becomes more and more important [15].

For risk and toxicity assessment, the first step in screening nanomaterials is usually in vitro testing. For this purpose, various biochemical and biophysical assays for toxicity and viability testing of cells are available [16]. However, these current assays also include challenges due to interactions of optically active nanomaterials with colorimetric cytotoxicity tests, which can affect the measurement results. Therefore, test systems have to be carefully selected to achieve an accurate hazard and safety characterization [17–19]. Additionally, a careful consideration of experimental conditions is crucial to obtain reliable and realistic data sets and the cell type as well as realistic particle doses must be selected thoroughly to reflect the route of introduction and target organ of the nanoparticle [20, 21]. Beside the aspect of particle interference in marker-based assays, there remain some other general limitations of in vitro assays in nanotoxicology. These can include differences in the production and properties of tested nanomaterials and variations in the cell types that are used for testing. Another challenging issue are adequately standardized testing protocols for nanomaterials which are sufficiently verified by reference materials or interlaboratory validation. Hence, variations in experimental procedures may cause differences in measurement data and potentially even may lead to contradictory results [19, 22, 23]. These findings create a demand for new methods to analyse cellular responses to nanoparticles that are insensitive to interactions with the tested nanomaterial and are also suitable for combination with other biochemical analysis methods downstream [24, 25]. In addition, improved screening procedures are desirable, such as the determination of endpoints based on biophysical cell markers and the prospect enhancements in reliability, robustness and measurement speed.

Quantitative phase imaging (QPI) [26] provides in vitro assays with minimal material interference and light exposure for minimally invasive label-free imaging of biological samples in preclinical biomedical settings, which represent a particular advantage for quantifying effects of nanoparticles on cells and tissues. Digital holographic microscopy (DHM) [27] that is an interferometry-based variant of QPI allows assessment of biophysical parameters from DHM quantitative phase images, such as cellular dry mass, volume and refractive index as well as simplified image processing for extraction of cell morphology features [24, 28, 29], and quantitative monitoring of dynamic cell processes such as migration, proliferation, as well as apoptosis or necrosis is enabled [30, 31]. In earlier studies, DHM has been demonstrated as fast and reliable method which successfully contributed to various biomedical research areas [32, 33] including, e.g., blood analysis [34–37], assessment of pathological tissue [28] and in vitro toxicity testing [22, 24, 25]. In contrast to most common colorimetric cell viability and proliferation assays, where specific endpoints are determined that need to be defined prior the experiment, QPI with DHM facilitates continuous time-resolved long-term assessment which allows a variable extraction of endpoint data at any time point within the measurement period, even subsequently [22]. The label-free nature of DHM also eliminates problems with biological effects of dyes on cells.

This study addresses current limitations and challenges in standardized cytotoxicity testing and the assessment of nanomaterials by using DHM as a QPI-based in vitro assay to quantify polymeric nanocarrier effects. In comparative experiments that were performed within the EU Horizon 2020 project “Regulatory Science Framework for Nano(bio)material-based Medical Products and Devices (REFINE)” on human A549 lung epithelial cells at two collaborating laboratories (Biomedical Technology Center, University of Muenster, Germany; SINTEF Industry, Trondheim, Norway), we investigated the interlaboratory variability and performance of DHM in nanomaterial toxicity testing. At both participating laboratories, identical DHM instruments, developed for usage in biomedical laboratories, with stage-top incubator systems for QPI imaging of cells in 96-well plates were used. Experiments were conducted according to coordinated standard operation procedures (SOPs) with well-characterized nanoparticles. At both locations, A549 cells were incubated with the same concentration sets of poly(alkyl cyanoacrylate) (PACA) nanoparticles [38] for drug delivery, and cabazitaxel-loaded PACA nanoparticles [39, 40] for cancer treatment, and then observed by time-lapse DHM over a period of 12 h versus digitonin as cytotoxicity control and incubation with cell culture medium. The obtained DHM QPI image series were subsequently evaluated for changes in cellular dry mass development and morphology alterations as readouts for proliferation and cell viability, and the resulting measurement data were comparatively analysed to quantify the performance as well as the interlaboratory variability of the retrieved biophysical parameter sets.

## Material and methods

### Design and implementation of the comparative interlaboratory DHM study

**Fig. 1:**
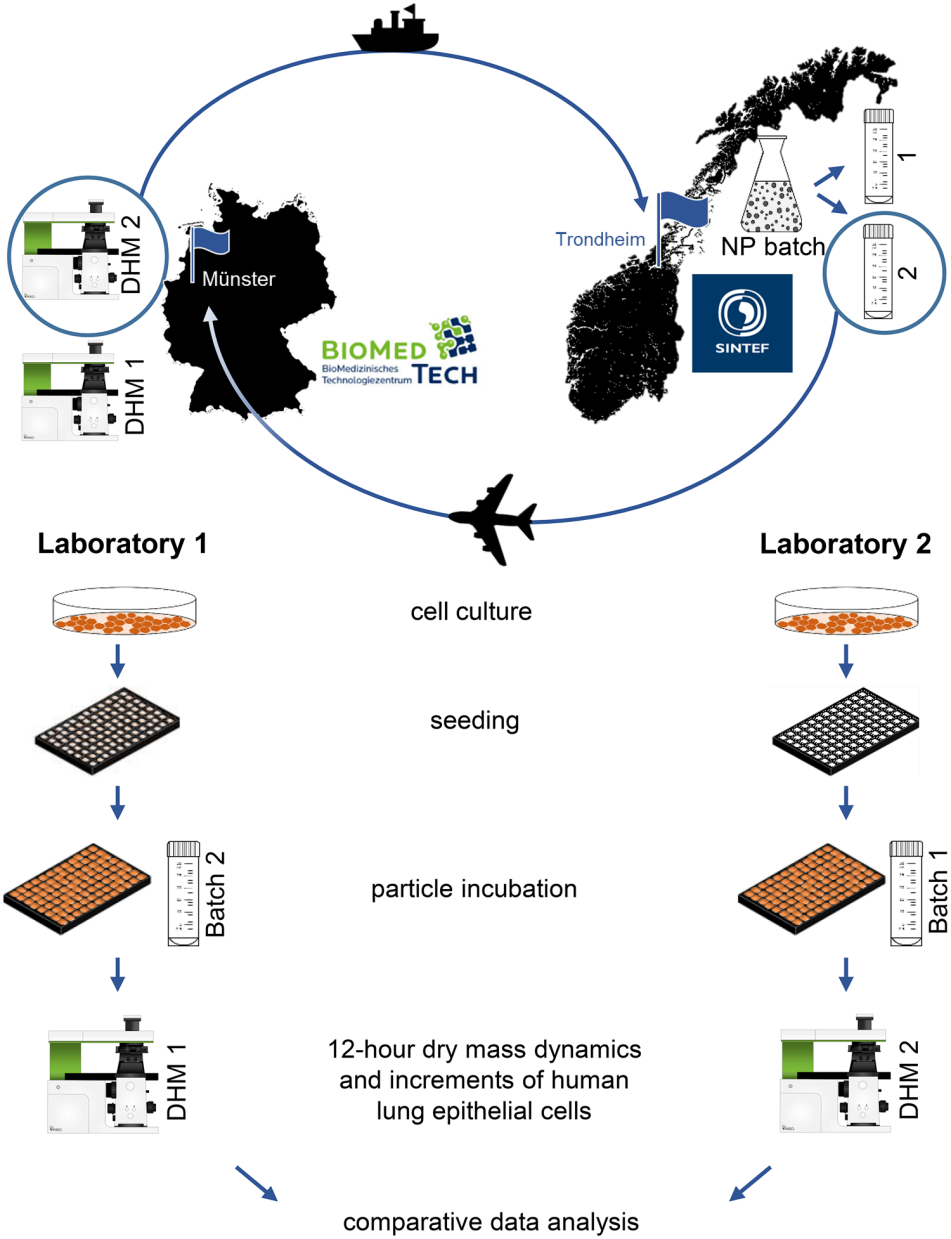
General organization and workflow of the interlaboratory variability evaluation of the label-free DHM toxicity assay. Polymeric nanoparticles were synthesized at laboratory 2 from which one batch was sent to partner laboratory 1. Two identical DHM systems were provided by laboratory 1 from which one device was shipped to laboratory 2. Each laboratory performed independently cell culture and DHM measurements on nanomaterial incubated cells and controls according to a collaborative elaborated SOP for retrieval of quantitative phase images every 60 min for 12 h from which the temporal dry mass development as well as the dry mass increment of the fraction of rounded cells after 12 h were determined. Data from both laboratories were comparatively evaluated

Figure 1 shows in an overview the general organization and workflow of the comparative interlaboratory study. Two collaborating laboratories, Biomedical Technology Center, University of Muenster, Germany (Laboratory 1), and SINTEF Industries, Trondheim, Norway (Laboratory 2), analysed the interlaboratory variability and performance of DHM for toxicity testing of well characterized polymeric nanomaterials by comparative experiments on A549 human lung epithelial cells. In order to achieve comparability of experimental data at both laboratories, identical conditions had to be established. For this reason, and due to the large distance between the two partner laboratories, the study design included not only the workflow but also the spatial transfer of materials and equipment. Nanomaterials for testing were synthesized and characterized at laboratory 2, from which one batch was sent to partner laboratory 1. Moreover, to minimize differences in the QPI measurements, two identical DHM systems, built for usage in biomedical laboratories, were provided by laboratory 1 from which one device was shipped to laboratory 2. In addition to materials and equipment management, a detailed SOP for performing the DHM-based assay was elaborated and shared between the partners. Based on the joint SOP, each laboratory independently performed cell culturing and DHM measurements on nanomaterial incubated cells. Cell culture handling was performed using each laboratory infrastructure, cell and nutrient as described in the “Cell preparation for DHM experiments” section. Experimental data achieved at both laboratories were evaluated by staff of laboratory 1 and analysed together by both research teams.

### Synthesis and characterization of polymeric nanocarriers

The polymeric nanoparticles were synthesized at laboratory 2 (SINTEF Industry, Trondheim, Norway) by emulsion polymerization from a water phase containing alkyl cyanoacrylate monomers and an aqueous phase containing hydrochloric acid and polyethylene glycol (PEG) surfactants and provided to laboratory 1. Tested materials were PACA particles [38] developed for drug delivery and cabazitaxel (cbz)-loaded PACA nanoparticles [39, 40] that were designed for cancer treatment. The particle diameter was determined by dynamic light scattering (DLS) and found for both unloaded and cbz-loaded PACAs, in the range of 134–140 nm with a narrow size distribution (polydispersity index (*PDI*) ≤ 0.13). Both particles displayed a slightly negative charge with a zeta potential of about −4.8 (PACA) and 5.5 mV (PACA cbz). Endotoxin levels were < 1 EU/mL.

### Cultivation of A549 lung epithelial cells

A549 lung epithelial cells (ATCC^®^ CCL-185, American Type Culture Collection, Manassas, VA, USA) were cultivated in both laboratories according to standard cell culture procedures in Dulbecco’s modified Eagle’s medium (DMEM, Sigma-Aldrich, St. Louis, MO, USA) supplemented with 10% foetal calf serum (FCS, PAN Biotech, Aidenbach, Germany), 1 mM pyruvate (Biochrom, Berlin, Germany) and 2 mM glutamine (Merck, Darmstadt, Germany) without antibiotics [41] and passaged twice a week. Mycoplasma contamination was controlled by qPCR, and cell culture A549 passages 5–30 were used for DHM QPI experiments.

### Cell preparation for DHM experiments

For DHM QPI time-lapse experiments, A549 cells were cultivated up to a confluence of 90%, harvested with trypsin/ EDTA (Sigma-Aldrich, St. Louis, MO, USA), pelleted at 330 × g for 5 min, resuspended into filtered cell culture medium and then seeded in 300 µL volume at a density of 20,000 cells/mL into black 96-well imaging plates (µ-Plate 96 Well Black, ibidi, Munich, Germany). Cell densities were determined automated in both laboratories. In laboratory 1, a label-free digital holography-based device (Fluidlab R-300, Anvajo, Dresden, Germany) was used, while in laboratory 2, an automated fluorescence-based cell counting device (Countess II, Invitrogen, Waltham, USA) was applied. Cells were incubated in 96-well imaging plates for 24 h at 37 °C and 5% CO_2_ and then incubated with nanoparticles and controls. The cell culture medium was replaced by filtered cell culture medium which included either the cytotoxicity control digitonin or the tested PACA nanoparticles. Digitonin (Sigma-Aldrich, St. Louis, MO, USA) was applied in concentrations of 0.1, 5.5, 20 and 140 µg/mL, while PACA and cbz-loaded PACA nanoparticles were tested at concentrations of 2, 8, 32 and 128 µg/mL. Afterwards, 96-well plates were transferred into the prepared incubation chamber on the DHM system (for a detailed description of the DHM setup see the “Utilized DHM systems and generation of QPI images” section).

### Utilized DHM systems and generation of QPI images

**Fig. 2:**
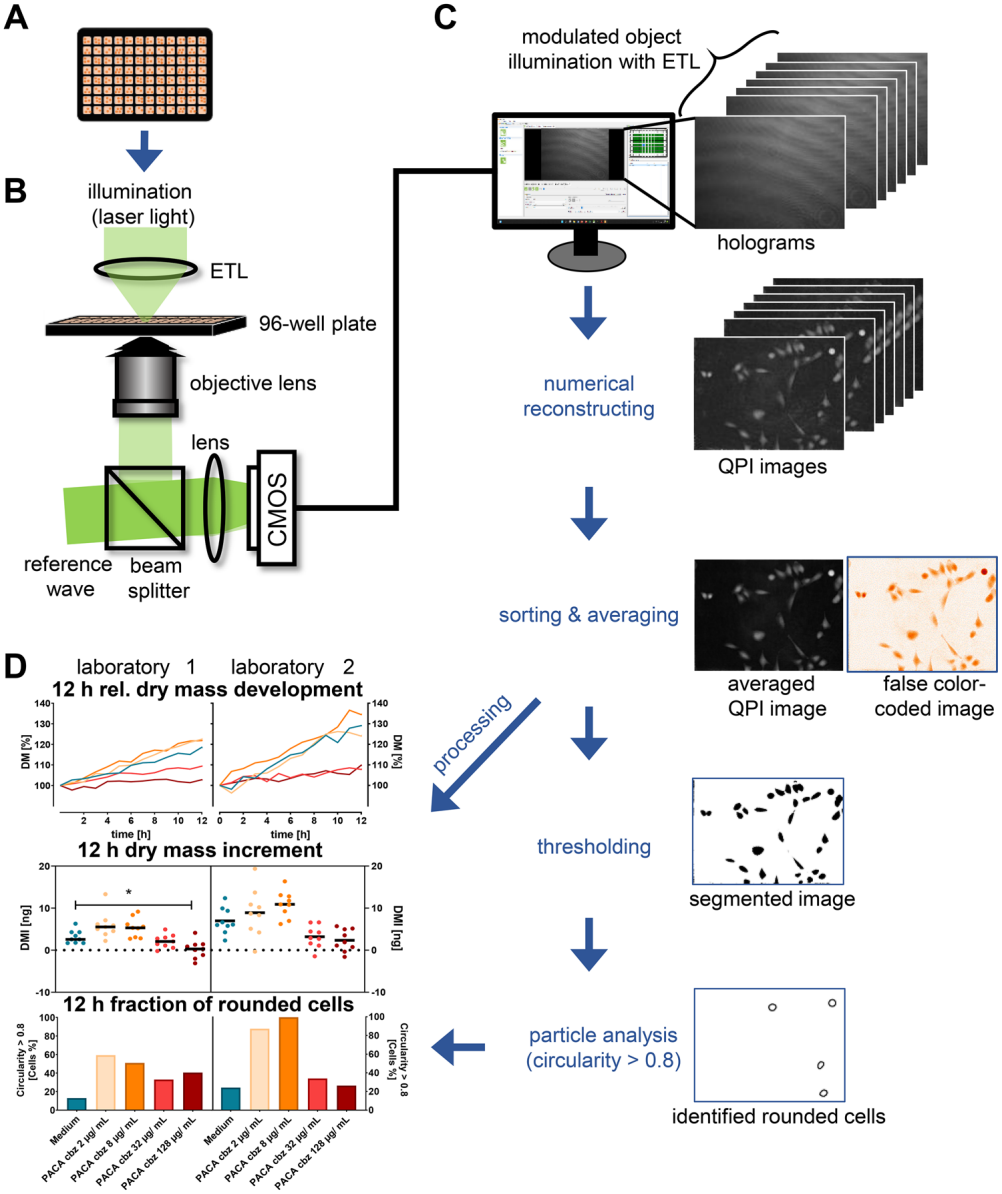
Experimental workflow of the DHM in vitro assay performed in both laboratories for cytotoxic effect quantification. Polymeric nanocarriers were tested in comparison to cytotoxicity and medium control. **A** A549 cells were seeded into 96-well imaging plates and incubated with PACA nanoparticles and controls. **B** Quantitative phase imaging was performed using an inverted research microscope equipped with an off-axis DHM module and a stage-top incubator. Sequences of 7 digital off-axis holograms were acquired with modulated object illumination via an electrically tunable lens (ETL) at *n* = 3 different measurement positions in one of the three wells per concentration every 60 min over a period of 12 h. **C** DHM QPI images were numerically reconstructed from the captured hologram sequences and subsequently averaged for each time point and position to reduce image disturbances caused by the coherence properties of the applied laser light. To quantify particle effects on cell morphology, QPI images were normalized, segmented based on thresholding and analysed for the morphology-related parameter circularity. A circularity threshold *C* > 0.8 was set to identify cells with spherical shape and was used to determine the fraction of rounded cells for each experiment after 12 h. **D** Temporal relative dry mass development (*DM*) and dry mass increments (*DMI*s) after 12 h (*DMI*) were determined. DM of cells was averaged from *N* = 3 measurements including *n* = 9 FOVs and normalized to the initial dry mass of the respective cell population. *DMI* values were plotted individually for or all *n* = 9 FOVs investigated in three independent experiments (*N* = 3). Twelve-hour fractions of rounded cell are provided as percentage fractions. Statistical significance of dry mass increments was analysed by multi-factorial analysis of variance; ***p < 0.005, **p < 0.01, *p < 0.05

The interlaboratory comparative evaluation experiment was conducted by applying two identical off-axis DHM systems, which were developed for the usage in the non-vibration isolated environment of biomedical laboratories, based on previously described concepts [22]. One of the devices was utilized for QPI experiments at laboratory 1 on-site, while the other one was shipped to laboratory 2. In short, the two DHM systems consisted of inverted Nikon Ts2R microscopes (Nikon, Tokyo, Japan) equipped with attached DHM modules [42] and motorized microscope stages (Märzhäuser, Wetzlar, Germany) for automated data acquisition and were capable for bright-field imaging and quantitative phase imaging (QPI) of living cells (Fig. 2B). A stage-top incubator chamber with heating system (K-frame heating system, ibidi, Munich, Germany) and gas incubation system (K-frame gas control system, ibidi, Munich, Germany) allowed time-lapse investigations of living cells in 5% CO_2_ atmosphere at physiological temperature (37 °C). Coherent light sources for the recording of digital holograms were fibre-coupled solid-state lasers (Cobolt 06-DPL, *λ* = 532 nm, Cobolt AB, Solna, Sweden). DHM time-lapse imaging for quantification of cytotoxicity caused by organic nanoparticles was performed as illustrated in Fig. 2 and has been described with technical details in a previously published study [22]. Digital off-axis holograms of cells were recorded with a complementary metal–oxide–semiconductor (CMOS) sensor (UI-3260CP-M-GL, IDS GmbH, Obersulm, Germany) using a 20 × microscope lens (Nikon Plan 20x/0.4, Nikon, Japan) as illustrated in Fig. 2B. For each measurement and time point, one bright-field image and 7 digital off-axis holograms were captured with exposure times in millisecond or sub-millisecond range while the object illumination wave was modulated by an electrically tuneable lens [43]. Subsequent processing of the acquired holograms was performed as illustrated in Fig. 2C. First, quantitative phase images were reconstructed from each series of digitally captured holograms numerically utilizing a previously described variant of the Fourier transformation method [44] and optional numerical refocussing [45]. Then, QPI images for every position and time point were averaged to reduce coherence induced image disturbances [43]. Prior the experimental investigations, the QPI image quality of each DHM system was analysed utilizing a 3D-printed phase test chart as shown in supplementary material, Fig. S1 [46].

### DHM assay conduction and QPI data evaluation for quantification of nanocarrier effects on cells

The A549 cells seeded in 96-well imaging plates (Fig. 2A) were incubated with organic nanoparticles and placed in the stage-top incubator of the DHM imaging systems (Fig. 2B). Bright-field images and holograms were recorded every 60 min for 12 h at each observed field of view (FOV). In each DHM experiment, three wells per concentration (*n* = 3) were observed, while in each well, an individually selected represented FOV was measured. Experiments were repeated independently three times (*N* = 3) with different cell passage numbers in each laboratory. From the DHM QPI image data sets acquired at the two laboratories (Fig. 2C), dry mass development and increment data, as well as the circularity, a single-cell morphological related parameter, were calculated and used to quantify the cytotoxicity of the tested nanomaterials (Fig. 2D). The temporal dry mass development and the dry mass increment of the entire cell population within the FOV after 12 h were determined from the cell induced averaged phase shift [47] as described previously [22]:

1 $$dm=\frac{\lambda }{2\pi \alpha }\Delta \overline{\varphi }{S }_{\mathrm{FOV}}$$

In Eq. 1, the parameter $\Delta \overline{\varphi }$ represents the mean phase shift within the observed field of view *S*_FOV_ (450 µm × 338 µm for both utilized DHM systems) while *λ* = 532 nm represents the light wavelength of the utilized laser. For the specific refractive index increment, which relates the phase shift to the intracellular protein content, a value of *α* = 0.19 × 10^−3^ mm^3^/g was assumed [48–50]. In order to avoid variabilities due to different cell numbers at the beginning of the measurement, the dry mass (*dm*) was normalized to relative dry mass (*DM*) development with respect to *t* = 0 and dry mass increments (*DMI*s) after 12 h were calculated in relation to the values as *t* = 0, respectively:

2 $$DMI={dm}_{t=12\mathrm{h}}-{dm}_{t=0\mathrm{h}}$$

A morphological event that can be observed for both dying adherent cells, and the response to cytostatic drugs, is the rounding of cells and detachment from the substrate. To assess these morphological alterations, the fraction of cells with a spherical morphology was quantified. Therefore, individual cells in DHM QPI images after 12 h were threshold-based segmented utilizing the freely available software *ImageJ* version 1.52 s [51]. Subsequently, the *particle analysis* plugin of *ImageJ* was applied to extract the morphology-related parameter circularity:

3 $$C=4\pi \frac{[{S}_{\mathrm{cell}}]}{{[P]}^{2}}$$

In Eq. (3), *C* represented the circularity of a segmented single cell within a QPI image, with the surface area *S*_cell_, and the corresponding perimeter *P.* Cells with a circularity *C* > 0.8 were considered as detached cells with spherical morphology. During cell segmentation, a size threshold *S*_cell_ > 350 µm^2^ was applied excluding cell debris from the data evaluation. Based on Eq. (3), for each experiment, the fraction of rounded cells was determined by dividing the number of cells with *C* > 0.8 through the total number of identified cells in the analysed FOVs after 12 h of incubation with controls and organic nanocarriers.

### Statistical analysis

All data were produced in *N* = 3 independent conducted experiments, while in each experiment, *n* = 3 FOVs were evaluated for each of the two participating laboratories. For the dry mass increments, statistical significance was calculated using GraphPad Prism version 8.3.0 using multi-factorial analysis of variance and significance levels were given as *p* < 0.005 (***), *p* < 0.01 (**) and *p* < 0.05 (*).

## Results

In a comparative study, we investigated the interlaboratory variability and performance of DHM in nanomaterial toxicity testing. To quantify the effects on cells upon incubation with nanocarriers and controls, two identical QPI instruments were established and implemented at each of the two participating partner laboratories. DHM QPI images of A549 lung epithelial cells were analysed qualitatively based on changes in cell growth and morphology at both laboratories. Data were obtained from three independent experiments (*N* = 3), each with *n* = 3 individually continuously observed FOVs. One independent experiment consisted of *n* = 3 FOVs of each: cell culture medium control and cytotoxicity control digitonin, as well as all PACA and PACA cbz concentrations. The corresponding cell culture medium control was compared to the results for digitonin and the two PACA particles. Relative dry mass development of the cell populations in the FOVs was determined to analyse the temporal response of cell proliferation compared to controls. Each data point represents the mean dry mass of the cells of *N* = 3 measurements, normed to the initial dry mass of each cell population. As an endpoint measurement, the dry mass increment (*DMI*) after 12 h was calculated. *DMI* values show the increments in nanograms from all *n* = 9 FOVs produced in three independent biological experiments (*N* = 3) at each of the two facilities. Since rounded cells are seen as an indicator of detached cells, cell circularity was determined and a circularity threshold *C* > 0.8 was set to determine the fraction of cells with spherical shape after 12 h in the FOVs.

**Fig. 3:**
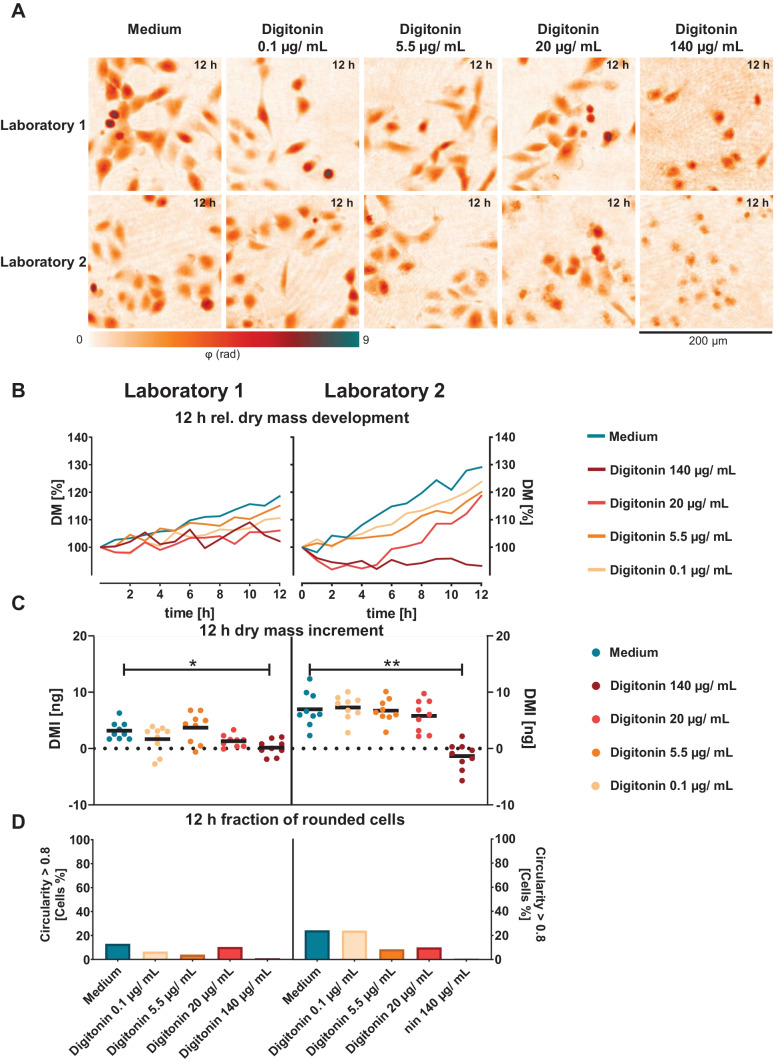
DHM QPI of A549 lung epithelial cells incubated with cell culture medium and cytotoxicity control digitonin. **A** Representative false color-coded QPI images of cells treated with cell culture medium control and cytotoxicity control digitonin in different concentrations after 12 h. **B** Temporal *DM* development of cell populations in the FOV retrieved for medium control and digitonin. Each data point represents the average dry mass of *n* = 9 FOVs (*n* = 3 FOVs per independent experiment) in *N* = 3 independent experiments for each laboratory. **C** Mean increment of the cell population dry mass (*DMI*) in the FOVs after 12 h. **D** Relative fraction of cells in the FOVs with circularity *C* > 0.8 after 12 h

First, the effects of the cytotoxicity control digitonin were quantified in comparison with the medium control. Figure 3A shows representative false color-coded DHM QPI images of A549 lung epithelial cells incubated with cell culture medium and cytotoxicity control digitonin in different concentrations. Corresponding sets of captured bright-field images for the same FOVs are provided in Fig. S2A in the supplementary materials. Images of the medium control for both laboratories showed viable cells within the analysed area. It can be observed that the A549 cells in laboratory 1 showed a more elongated growth, whereas the morphology of the cells in laboratory 2 was more epithelial and isoprismatic. Cell numbers in laboratory 2 were slightly higher than for laboratory 1 at the start of the measurement and after 24 h of growth. Cells incubated with 0.1 and 5.5 µg/mL digitonin proliferated to a similar extent as medium control cells for both laboratories. Digitonin (20 µg/mL) caused cell debris in a few cells, more for laboratory 2 than for laboratory 1. Digitonin (140 µg/mL) caused cell degradation and debris in both laboratories. In Fig. 3B, temporal *DM* development of cell populations in the FOVs is shown. For both laboratories, cell lines showed a continuous increasing *DM* development for the cell culture medium control during the 12 h observation period. In laboratory 1, the cells with lower digitonin concentrations 0.1 and 5.5 µg/mL showed a similar but lower *DM* development as the corresponding medium controls. For higher digitonin concentrations (20 µg/mL), cells showed moderate proliferation. Cells incubated with the highest digitonin concentration (140 µg/mL) showed a low and discontinuously *DM* development. In laboratory 2, the temporal *DM* developed in a more rapid manner, in contrast to laboratory 1, where A549 cell populations showed a moderate developing *DM* during the 12-h period. Cells incubated with 0.1 and 5.5 µg/mL digitonin showed a similar and lower *DM* development as the medium control; here, similar to laboratory, 1.20 µg/mL digitonin caused a decreasing *DM* development over the first 5 h followed by a distinct exponential proliferation up to 12 h. While cells incubated with 140 µg/mL digitonin, a decreasing *DM* development can be observed. The observations on the *DM* development in Fig. 3B are also reflected in the *DMI*s of the cell populations (Fig. 3C). In laboratory 1, the mean *DMI* of cell culture medium–treated cells across the nine observed FOVs was determined to 3.2 ± 1.6 ng. For incubation with 0.1 µg/mL digitonin, the *DMI* of cell populations were lower after 12 h than for the cell populations of the medium control (1.7 ± 2.4 ng). *DMI*s for cells treated with 5.5 µg/mL digitonin were as similar as *DMI*s of medium control cells (3.7 ± 2.7 ng). At 20 µg/mL digitonin, the mean *DMI* is lower but no significant differences compared to the medium control could be detected (1.3 ± 1.1 ng). The highest digitonin concentration of 140 µg/mL caused significant effects compared to medium incubated cells. Cells displayed a significantly (*p < 0.05) differing *DMI* of 0.2 ± 1.4 ng. In laboratory 2, a mean *DMI* for medium control cells of 7.0 ± 3.1 ng was obtained. For incubation with 0.1 µg/mL and 5.5 µg/mL digitonin, *DMI*s of cell populations were as similar after 12 h as for the cell populations of the medium control (7.3 ± 2.2 ng for 0.1 µg/mL and 6.7 ± 2.1 ng for 5.5 µg/mL). With digitonin concentrations of 20 µg/mL, a slightly lower *DMI* than cell populations of the medium control can be observed (5.8 ± 2.8 ng). Digitonin concentrations of 140 µg/mL caused a highly significant (**p < 0.01) difference in *DMI* (−1.3 ± 2.4 ng) compared to medium-treated cells. Figure 3 D shows the fractions of rounded cells in the investigated FOVs for medium and digitonin treated cells after 12 h that were determined based on the morphology-related parameter circularity *C* where *C* > 0.8 was set to identify cells with spherical shape. For laboratory 1, 13% of the measured cells in the medium control showed a circularity *C* > 0.8. Among the cells treated with 0.1 µg/mL digitonin, a fraction of 6% with circular shape was detected, while with 5.5 µg/mL digitonin, the fraction of rounded cells decreased to 4%. At 20 µg/mL of the cytotoxicity control, 10% of the incubated cells were found to have *C* > 0.8, compared to the highest concentration of 140 µg/mL where only a small fraction of cells presented a spherical morphological phenotype (1%). In contrast, medium control cells of laboratory 2 showed a higher fraction of cells with *C* > 0.8 than at laboratory 1 (24%). Apart from the lowest digitonin concentration of 0.1 µg/mL (23%), the higher digitonin concentrations showed a similar decrease in the relative number of rounded cells compared to the medium control as in laboratory 1. For 5.5 µg/mL digitonin, 8% of the cells showed a spherical phenotype. The fraction of rounded cells increased to 10% after incubation with 20 µg/mL digitonin. For the highest concentration of 140 µg/mL, only 1% of the analysed cells was found to be spherical with *C* > 0.8 as digitonin affected the cell membrane and caused irregular cell debris which considerably reduced the fraction of intact cells in the analysed FOVs after 12 h of incubation.

**Fig. 4:**
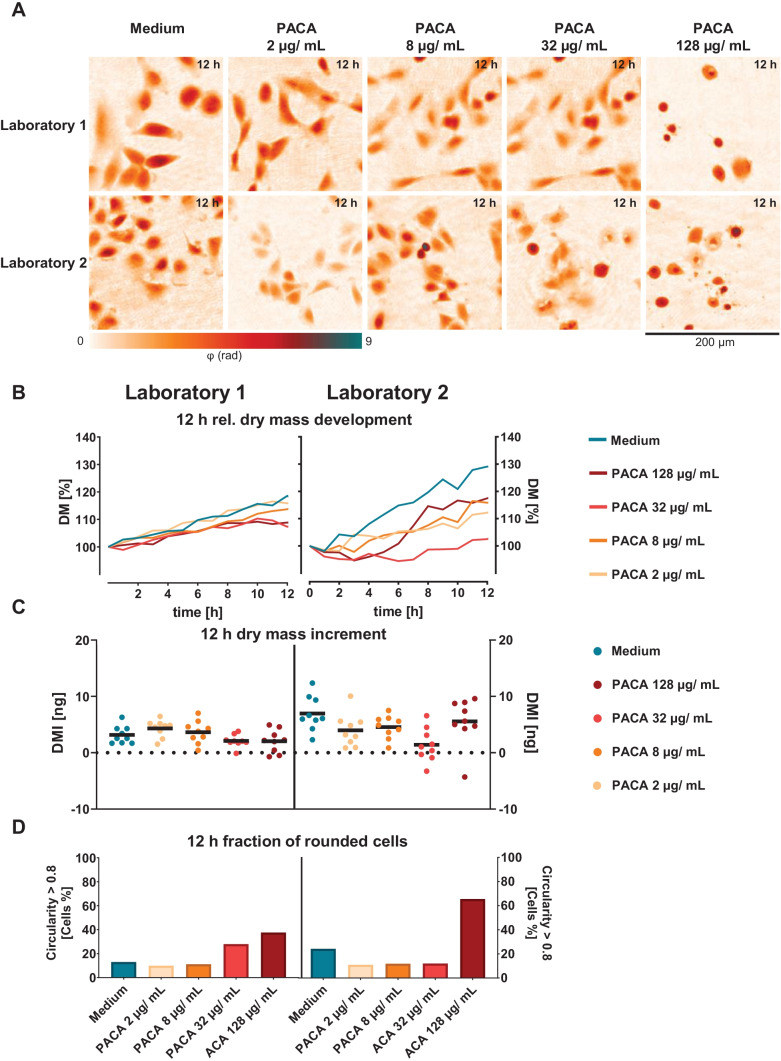
DHM QPI of A549 lung epithelial cells incubated with medium and PACA nanoparticles. **A** Representative false color-coded QPI images of cells treated with cell culture medium control and polymeric nanocarriers in different concentrations after 12 h. **B** Temporal *DM* development of cell populations in the FOV retrieved from DHM time-lapse measurements for medium control and PACA particles. Each data point represents the mean of dry mass of *n* = 9 FOVs (*n* = 3 FOVs per independent experiment) in *N* = 3 independent experiments for each laboratory. **C** Mean increment of the cell population dry mass (*DMI*) in the FOVs after 12 h. **D** Relative fraction of cells in the FOVs with *C* > 0.8 after 12 h

In a next step, the cytotoxic effects of PACA nanoparticles were quantified. Figure 4A depicts representative false color-coded DHM QPI images of A549 lung epithelial cells retrieved at both laboratories after treatment with PACA nanoparticles for 12 h and the corresponding cell culture medium control. Corresponding sets of captured bright-field images for the same FOVs are provided in Fig. S2B in the supplementary material. QPI images for the medium control showed viable cell populations at both laboratories. After incubation with 2 and 8 µg/mL of PACA nanocarriers, cells proliferated to a similar extent as medium control cells for both laboratories and no visible changes in cell morphology were observed. In contrast, for 32 µg/mL of PACA particles, cells detached and deformed at laboratory 2, while at laboratory 1, no effects were observed for this particle concentration. A further increase of the concentration to 128 µg/mL PACA nanocarriers caused detached and deformed cells in both laboratories. Figure 4B shows the *DM* development of the cell populations in the FOV after treatment with the polymeric nanocarriers vs. the cell culture medium control during the entire observation period of 12 h. For laboratory 1, a linear *DM* development is observed for all PACA concentrations. A concentration of 2 µg/mL of polymeric nanocarriers caused similar cell proliferation and *DM* developments as for medium-treated cells. Incubation with 8 µg/mL caused a lower *DM* development than medium incubated cells. Cell populations incubated with 32 and 128 µg/mL of PACA nanoparticles caused a continuously but low increasing *DM* development. In laboratory 2, the *DM* development showed non-linear and partly discontinuous courses. Of polymeric nanocarriers, 2 µg/mL and 8 µg/mL led to a lower *DM* development than corresponding medium control cells. In contrast to laboratory 1, 32 µg/mL caused an even lower cell proliferation, and while first the *DM* development of cells treated with 128 µg/mL PACA showed a decrease, after 6 h of incubation, the *DM* curve displayed a positive development. The corresponding mean *DMI*s in the FOVs after 12 h are plotted in Fig. 4C. At laboratory 1, 2 µg/mL of polymeric nanocarriers caused higher increments as medium-treated cells (4.3 ± 1.5 ng). A PACA concentration of 8 µg/mL caused a similar mean *DMI* than the cells of the medium control with 3.7 ± 2.0 ng. 32 and 128 µg/mL of PACA nanoparticles caused lower mean *DMI* values compared to the medium control (2.1 ± 1.1 ng and 2.0 ± 2.0 ng). In laboratory 2, the *DMI* of cells treated with 2 µg/mL PACA particles was lower than the *DMI* of medium control cells and even lower compared to cells treated with the same nanoparticle concentration of laboratory 1 (4.0 ± 2.9 ng). Cell populations in laboratory 2 incubated with 8 µg/mL of PACA particles showed a lower *DMI* than medium-treated cells but a higher *DMI* than cells with 2 µg/mL of the particles 10.9 ± 3.2 ng. Of the polymeric nanocarriers, 32 µg/mL caused lower *DMI*s but no significant effects. This nanoparticle concentration led to a mean *DMI* of 1.1 ± 3.0 ng, whereas 128 µg/mL PACA caused a *DMI* of 5.6 ± 4.2 ng. Figure 4D shows the fraction of rounded cells indicating detached cells. Shown is the relative number of cells with a circularity *C* > 0.8 for cells treated with medium and PACA nanomaterials. In laboratory 1, among cells treated with 2 µg/mL of the polymeric nanocarriers, 10% of cells showed a circularity *C* > 0.8. For 8 µg/mL, the fraction of rounded cells was found similar for both laboratories. For 8 µg/mL, 11% of the cells demonstrated a spherical morphology. Almost 28% of the cells treated with 32 µg/mL of the nanomaterial showed the spherical shape. The highest concentration of PACA nanoparticles led to a strong increase of cells with *C* > 0.8 compared to the medium control cells. Of the cells, 38% presented a circular phenotype. In laboratory 2, 11% of cells showed a circular morphology. For 8 µg/mL, the fraction of cells with *C* > 0.8 increased to 12%. In contrast to laboratory 1, only 12% of the cells treated with 32 µg/mL of the nanomaterial displayed a circular phenotype. High PACA concentrations led to a high fraction of spherical cells, compared to the medium control. Sixty-five percent of the cells were rounded. The results for both laboratories are in accordance with the QPI images shown in Fig. 4A, where many rounded and detached cells can be seen at the highest PACA concentrations. Additionally, it is noticeable that the fraction of circular cells in laboratory 2 is in line with the *DMI* trend for the different PACA concentrations.

**Fig. 5:**
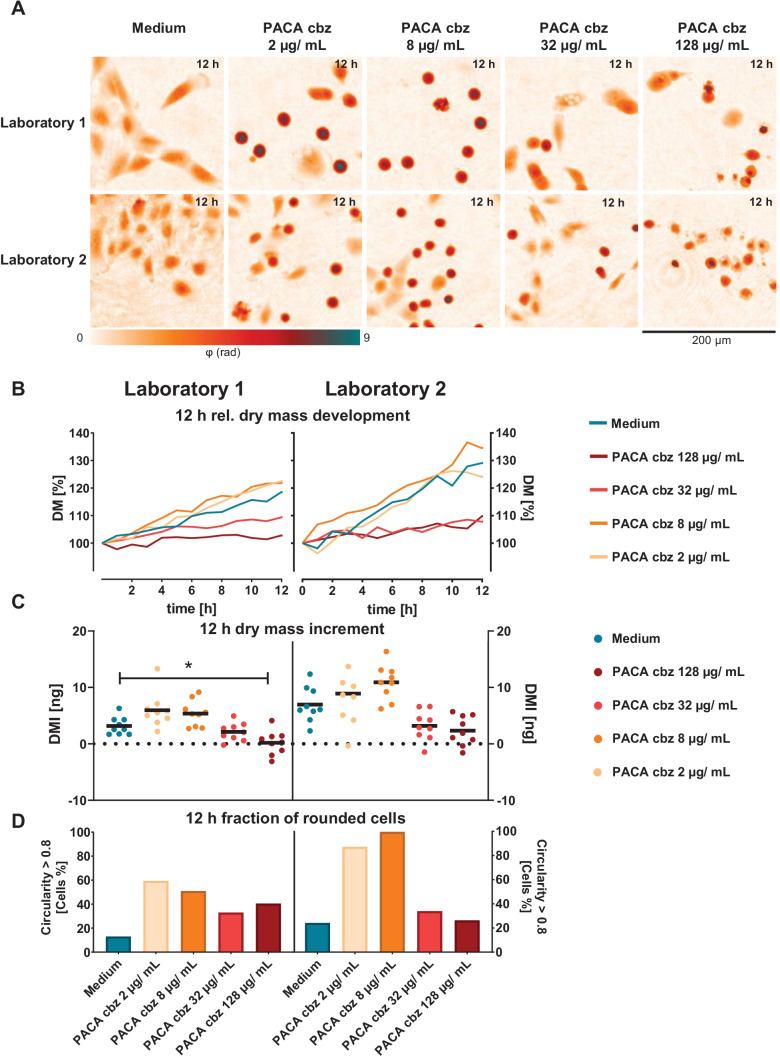
DHM QPI of A549 lung epithelial cells incubated with medium and PACA cbz nanoparticles. **A** Representative false color-coded QPI images of cells treated with cell culture medium control and polymeric nanocarriers loaded with cbz in different concentrations. **B** Temporal *DM* development of cell populations in the FOV retrieved from DHM time-lapse measurements for medium control and PACA cbz particles. Each data point represents the mean of dry mass of *n* = 9 FOVs (*n* = 3 FOVs per independent experiment) in *N* = 3 independent experiments for each laboratory. **C** Mean increment of the cell population dry mass (*DMI*) in the FOVs after 12 h. **D** Relative fraction of cells in the FOVs with *C* > 0.8 after 12 h

Finally, the cytotoxicity of cbz-loaded PACA nanocarriers was tested. Results are shown in Fig. 5. Figure 5A presents representative false color-coded DHM QPI images of A549 lung epithelial cells treated with PACA cbz nanoparticles, and cell culture medium controls are shown. Corresponding sets of captured bright-field images for the same FOVs are provided in Figure S2C in the supplementary materials. Medium control QPI images for both laboratories show proliferating cell populations. Cells incubated with PACA cbz nanocarriers caused visible changes in cell morphology and inhibited proliferation compared to medium control cells for both laboratories could be detected. Concentrations of 2 and 8 µg/mL of PACA cbz particles caused detached cells, whereas 32 and 128 µg/mL of cbz-loaded nanocarriers led to detached and to deformed cells. The 12-h *DM* development of the cell populations incubated with PACA cbz and cell culture medium control is presented in Fig. 5B. For laboratory 1, a continuously increasing *DM* development in a higher extent as the medium control can be observed for cells incubated with 2 and 8 µg/mL of the polymeric nanocarriers loaded with cbz. *DM* developments of cell populations treated with 32 µg/mL PACA cbz showed lower courses than *DM* curves of the medium control. The highest concentration of loaded nanocarriers (128 µg/mL) led to the lowest *DM* development. In laboratory 2, for cells incubated with 2 and 8 µg/mL of the polymeric nanocarriers loaded with cbz, *DM*s developed in a similar extent or higher as *DM*s of medium control cells similar in laboratory 1. Cell populations treated with 32 µg/mL PACA cbz showed *DM* developments which are lower than *DM* curves of the medium control and similar to laboratory 1. Of loaded nanocarriers, 128 µg/mL caused low *DM* developments, similar to 32 µg/mL. The mean *DMI*s in the FOVs after 12 h of incubation are presented in Fig. 5C. In laboratory 1, *DMI*s were higher than *DMI*s of the medium control with 6.0 ± 3.1 ng for 2 µg/mL and 5.3 ± 2.3 ng for 8 µg/mL. Cells treated with 32 µg/mL of PACA cbz showed a *DMI* of 2.1 ± 1.6 ng, which is lower than the *DMI* of medium-treated cells. Of loaded nanocarriers, 128 µg/mL had significant effects (*p < 0.05) on the cell populations (0.2 ± 2.1 ng). In laboratory 2, the mean *DMI* for 2 µg/mL of PACA cbz was higher than the *DMI* of medium-incubated cells (8.9 ± 5.7 ng); for cells treated with 8 µg/mL, it was even higher with 10.9 ± 3.2 ng. Of PACA cbz, 32 µg/mL led to a lower but not significant *DMI* of 3.2 ± 2.6 ng. Although a low *DMI* was also observed as in laboratory 1, 128 µg/mL PACA cbz had no significant effect on cell populations of laboratory 2 (2.3 ± 2.6 ng). The results from the morphological analysis of individual cells of the cell parameter circularity which indicates detached cells are shown in Fig. 5D, where the fraction of cells with a circularity *C* > 0.8 for cells treated with medium and PACA cbz is presented. Graphs in Fig. 5D and the corresponding QPI images in Fig. 5A show that for both laboratories, considerably more cells could be detected that exhibited a circular morphology, when incubated with PACA cbz particles than when incubated with medium only. It is remarkable that the results on the number of circular cells agree with the *DMI*s; this concurrence is especially pronounced for laboratory 2. For PACA cbz concentrations with a high *DMI*, a high number of rounded cells could also be detected. Even for the lowest PACA cbz concentration (2 µg/mL), for laboratory 1, more than half of the cells (59%) were rounded and even more (87%) in laboratory 2. Among cells incubated with 8 µg/mL of the loaded nanocarriers, less cells of laboratory 1 showed a circular shape (51%), whereas in laboratory 2, almost all cells presented a circularity *C* > 0.8 (99%). Higher PACA cbz concentrations did not lead to an even higher number of spherical cells. For 32 µg/mL of the nanocarriers loaded with cbz, similar numbers of rounded cells could be detected for laboratories 1 and 2 (33% and 34%). A concentration of 128 µg/mL PACA cbz caused a circulation of 40% of the cells in laboratory 1; in laboratory 2, it was about a quarter of all cells (26%).

## Discussion

In our study, we investigated the interlaboratory use and variability of DHM based QPI for assessment of toxic effects caused by polymeric nanoparticles. QPI data were acquired at two laboratories located at different places in Europe, utilizing two identical DHM QPI instruments (Fig. 1). The two DHM systems were set up in both interlaboratory partner institutions, and nanomaterials of the same batch were distributed. To evaluate requirements for standardization and applicability in second party laboratories, using collaborative elaborated SOPs, A549 cells were incubated with controls and different concentrations of nanoparticles and observed in 96-well plates in a stage-top incubator by automated time-lapse DHM for 12 h (Fig. 2A, B). The QPI image data sets retrieved from the DHM time-lapse assay were analysed for the temporal dry mass development, as well as the dry mass increment and the morphology-related parameter circularity that indicated rounded cells with a spherical shape (Fig. 2C). The biophysical data sets obtained at both laboratories were comparatively evaluated (Fig. 2D) and jointly analysed by both participating research teams.

First, the performance of the DHM QPI assay in each laboratory was analysed by investigations on the impact of different concentrations of the cytotoxic control agent digitonin vs. the incubation with cell culture medium only (Fig. 3). Subsequently, the impact of two well characterized PACA and cbz-loaded PACA nanomaterials was determined (Figs. 4 and 5).

Growth rates of A549 lung epithelial cells for controls in cell culture medium were slightly higher at laboratory 2 as visible from the *DM* development in Fig. 3B. However, as evident for the corresponding *DMIs* in Fig. 3C, sensitivity of the A549 cell line to digitonin as cytotoxicity control was equivalent with the highest concentration of 140 µg/mL causing significant effects at both laboratories. Such variations in cell culture laboratories are common and likely originate from handling of cells and cell counting as described in other interlaboratory comparison studies before [52]. From Fig. 3C, it is also evident that 12-h *DMIs* of A549 cells incubated with the cell culture medium control and the lower digitonin concentrations up to 20 µg/mL, which did not cause a significant cytotoxic effect, were generally higher at laboratory 2. The findings described above in Fig. 3 on the growth rates of A549 cells in the two laboratories are in accordance with the appearance of cell densities in the recorded QPI image sequences after 12 h (Fig. 3A).

The more rapid growth rate of A549 cells in the cell culture medium control at laboratory 2, in comparison to laboratory 1 visible in Figs. 3B, 4B and 5B, may be explained by higher initial cell numbers in laboratory 2 and dry mass values observed for laboratory 2. As the same absolute cell numbers were seeded in both laboratories according to the applied SOP, a possible reason for these deviations may be that due to the infrastructures of participation laboratories, different cell counting devices were applied (see the “Cell preparation for DHM experiments” section). While laboratory 1 used a holography-based automated cell counter system, in laboratory 2, an automated cell counter relying on trypan blue staining was utilized.

Additional causes for *DMI* variabilities between the two laboratories (Fig. 3B) could have been differences in laboratory equipment or staff specific differences in procedures during cell culturing. The mentioned explanation is supported by the slightly different morphology of the A549 cells that had an influence on the growth, visible in Figs. 3A, 4A and 5A and corresponding correlative recorded bright-field images (Supplementary materials Figure S1) [53]. In contrast to this finding, the fractions of rounded cells with a circularity *C* > 0.8 after 12 h of incubation with cell culture medium control were comparable between the two laboratories as visible in Fig. 3D.

The slight variabilities in growth rates and morphology of in vitro cell cultures as evident in Fig. 3B may be caused by cells from different passage numbers [5–30] that were used in the experiments. Similar effects were described in an earlier study on human colorectal adenocarcinoma cells (Caco-2), where increased proliferation was observed for higher passage numbers and extended cultivation duration [54]. Additionally, shifts of cellular epigenetics have been reported over time in cell lines used for cytotoxicity studies, which were cultivated for prolonged times [55]. Precise synchronization of cell models may reduce differences between partner laboratories. However, in vitro cell culture variability may also be influenced by the specific charges of the applied cell culture sera [56], which typically exhibit considerable variations in composition and content [57]. These aspects underline the importance of standardization to achieve highly comparable and reproducible results, as already small variations in experimental procedures can lead to detectable differences. Thus, improved alignment of cell lines, sera and cell counting tools prospects a further enhancement of quality and comparability of results in DHM cytotoxicity studies.

Particle-induced cytotoxicity can also be determined by other label-free methods using confluent cell layers, such as measurement of transepithelial electrical resistance (TEER) [58], with limitations compared to DHM concerning costs, maximum sample numbers and sensitivity to detect morphology changes, or histogram-based evaluation of quantitative DHM phase contrast images [59]. An advantage of these methods for the toxicity assessment is that they do not rely on cell growth rate or dry mass increment, which can depend on specific cell culture conditions but only partly reflect the characteristics of proliferating cells. To explain inhibition of proliferation or cell death after incubation with the nanoparticles, cells were investigated in sub-confluent layers within their main growth phase. In confluent layers, cells enter into a stationary phase with low proliferation and may detach independently from nanoparticle incubation [60].

The mild detergent digitonin is commonly used as cytotoxicity control for in vitro cell culture assays due to its straight-forward laboratory handling and low human toxicity [61, 62]. We have reported earlier on the application of digitonin for DHM QPI cytotoxicity assay and found significant toxicity at 32 µg/mL in RAW 264.7 macrophages and 64 µg/mL in NIH-3T3 fibroblasts, NRK-52E kidney epithelial cells and RLE-6TN lung epithelial cells [22]. In this study, digitonin was confirmed appropriate as non-particle cytotoxicity control for interlaboratory comparison DHM QPI experiments conducted with A549 lung epithelial cells, as both partner laboratories quantified significant reduction of *DMI* at 140 µg/mL applied (Fig. 3C). Also, the fraction of rounded cells after 12 h of incubation was found to be reduced in a concentration-dependent manner in both laboratories (Fig. 3D). Taken together, the sensitivity of the A549 cell line to cytotoxic digitonin was independently confirmed in both laboratories with individual DHM systems.

Suitable controls for (nano)-cytotoxicological investigations are a central topic for many biochemical and biophysical in vitro assays. A study investigating a DHM-based in vitro cytotoxicity assay proposed mercury dichloride (HgCl_2_) suitable as non-particle cytotoxicity control for QPI experiments [24]. However, considering the human hazard of HgCL_2_, digitonin appears to be a potent alternative as cytotoxicity control for DHM experiments. A further harmonization and validation of assay procedures, as performed in this study for organic nanomaterial testing, could be achieved in the future by implementation of standard control materials, ideally as nanosized calibration materials [63]. Nanomaterial-based calibration standards would ideally have the property of high stability accompanied with known toxicity mechanisms. For example, amine-modified polystyrene nanoparticles could be promising candidates for this purpose due to their know cytotoxicity and characteristics [64].

The PACA nanoparticles caused no significant effect on 12-h *DMIs* in the A549 lung epithelial cells in both laboratories (Fig. 4C) which is in agreement with similar changes in morphology with increasing nanoparticle concentrations in Fig. 4A. The highest empty PACA concentration of 128 µg/mL caused detachment and lysis of cells, visible as smaller and high phase contrast cells in QPI images (Figs. 4A). The quantification of rounded or spherical cells confirmed this finding (Fig. 4D). For the highest concentration at laboratory 2, more than half of all cells represented a circularity *C* > 0.8, which can be interpreted as an indicator for cell detachment and death. The morphological event of cell rounding and detachment of cells from the substrate is described as an early event of cell death and impaired mitosis, but cells also round up and detach for a short time during mitosis [65, 66]. These observations on PACA nanoparticle effects are in line with published biochemical assay studies as well as with an earlier QPI analysis of PACA nanoparticle effects on other cell types in vitro [22, 39].

Laboratory 1 detected a significant reduction of A549 *DMI* at a concentration of 128 µg/mL PACA cbz nanoparticles, while in laboratory 2, none of the applied concentrations caused a significant *DMI* reduction (Fig. 5C). An earlier QPI study revealed a significant effect on *DMI*s in macrophages and fibroblasts already at a concentration of 16 µg/mL of PACA cbz nanoparticles [22]. Main differences to the toxicity testing experiments reported here are shorter incubation times and that a lower number of FOVs per experiment were evaluated, which may explain the lower statistical significance of the observed effects (Fig. 5C). In a previous study with four different cell lines, toxicity-related dry mass changes quantified with DHM after 24 h showed a similar statistical significance as a complementary performed WST-8 cell viability assay [22]. This indicates that statistical significance levels in the performed DHM assay can be expected in similar ranges as in dye-based in vitro cytotoxicity assays. Furthermore, the significant effects of PACA particles and cbz on cells observed with DHM are comparable to reference methods applied in earlier studies. In a trypan blue exclusion assay, cbz-induced effects on A549 cells from 1 µg/mL [67]. In another study, IC50 values for empty PACA particles in different cell lines were determined with a CellTiter-Glo^®^ assay, ranging from 18 µg/mL (OVCAR-3 cells) to over 300 µg/mL (DU 145 cells) [68]. Cbz-loaded PACA nanoparticles caused increased detachment and rounding of A549 lung epithelial cells in comparison to the cell culture medium control starting at 2 µg/mL at both laboratories. This effect was highly reproducible across all analysed FOVs and experiments (Fig. 5A). From literature, alterations of cell morphology can be expected for the active pharmaceutical ingredient-loaded organic nanocarrier, as cabazitaxel is a taxane group cytostatic drug [69], causing disruption of microtubule functions and arrest cells in G2/M phase [67, 69, 70]. The quantification of the fraction of rounded cells in the FOV after 12 h of incubation in Fig. 5D for cbz-loaded PACA revealed a laboratory-independent and reproducible increase of rounded cells at the lower concentrations of 2 and 8 µg/mL in comparison to the cell culture medium control. Note that at higher concentrations of cbz-loaded PACA nanoparticles (Fig. 5D, 128 µg/mL), unspecific cell death due to nanocarrier degradation products becomes prominent and the fraction of rounded cells is reduced (Fig. 5D). This morphological response can be explained by more granulated round cells with thin extensions (see bright-field images in Fig. S2C). PACA nanocarriers are usually degraded within a few hours, depending on the length of the alkyl side chain of the PACA forming the nanospheres. This results in the degradation products alkyl alcohol and poly(cyanoacrylic acid) or oligomers of PACA [38]. In Figs. 4 and 5, we observed effects on cell morphology of cabazitaxel-loaded PACA nanoparticles at the lower concentrations (2 and 8 µg/mL), while empty PACA nanoparticles cause no effects at these concentrations. These findings are in line with results on four different cell lines tested in an earlier DHM-based study [22]. In the corresponding time-resolved QPI image stacks of the experiments with PACA cbz (data not shown), it is evident that cells already detach from the substrate after a few hours and remain detached until the end of the observation time. In an earlier study on A549 lung epithelial cells, pure cbz caused a significant twofold increase of cell death in comparison to the vehicle control starting from a concentration of 1 µg/mL after 12 h of incubation [67]. These previous observations are further emphasized by our results of the morphological examination in QPI images (Fig. 5A) and the analysis of fraction of rounded cells (Fig. 5D) in the lower PACA cbz concentrations.

Technical aspects that had to be considered in the interlaboratory evaluation of the digital holographic microscopy QPI assay were the individual performances of the two utilized DHM systems and the in situ conditions at each participation laboratory. The representatively shown DHM QPI images in Figs. 3A, 4A and 5A illustrate that in the non-vibration isolated environments of both laboratories, similar QPI image qualities and phase shift ranges of the investigated A549 lung epithelial cells were achieved. However, although DHM systems with identical components were used in the study, a spot test investigation with a durable phase test chart (Supplementary Fig. S1), provided by the Technical University of Warsaw, Poland, revealed slight differences in background phase noise. Moreover, individual parasitic interference patterns, caused by the coherence properties of the applied laser light, were evident. A typical origin of these disturbances are internal reflections which can be caused by the entire optical imaging path and depend on the individual system alignment. This underlines the importance of QPI system benchmarking as recently addressed in a study of National Institute of Standards and Technology (NIST) [71] and the demand for specifically tailored test charts for QPI system performance quantification, for example as proposed in [46], with respect to interlaboratory assay variability studies.

An additional notable aspect is that for the utilized laser light wavelength of 532 nm, the numerical aperture (NA) of 0.4 of the applied microscope lens results in a limited depth of field (DOF) which represents a potential error source for imaging of thicker cell structures that is objective specific and depends on the morphology of the investigated cell type. However, as in our study, no noticeable affectation of thicker cell structure in the quantitative phase images were observed (Figs. 3A, 4A and 5A); such effects can be assumed to be neglectable for the conducted experiments. Nevertheless, NA-related effects should be carefully considered when DHM data that were acquired by utilization of microscope lenses with different NAs are compared.

A further topic that had to be considered was that one of the utilized custom-built DHM systems had to be operated by scientists at laboratory 2 which required training by experienced operators from laboratory 1. Setup of the device included alignment and installation of the stage-top incubator and was performed by trained scientist from laboratory 1 within one working day. Considering the assay results in Figs. 3–5 and the findings in Supplementary Material Fig. S1, a key finding of this interlaboratory comparison study was that once setup and alignment of the DHM system was completed, cell culture handling and particle batches are the central points of standardization. Concerning the SOP applied for the DHM-based cytotoxicity assay this study, more extended EC_50_ determinations with increased concentration ranges of organic nanocarriers could further improve assay comparability and performance across interlaboratory partner institutions [22, 24]. Statistical evaluation of results also between laboratories, in addition to the analysis of results produced within a laboratory as performed herein, is commonly applied in interlaboratory comparison studies [52]. Even though we discussed the variabilities and accordance of QPI images and quantitative DHM results above, the data generated in the frame of this study is not fit for statistical analysis of between laboratory variance. Reasons for this observation in the data are the above discussed differences in the A549 cell culture at the two laboratories. Design of future studies may include necessary steps to allow for this kind of data evaluation by further harmonisation of procedures, devices and cell lines.

For processing of QPI images as illustrated in Fig. 2C, we applied in this study a threshold-based segmentation algorithm to evaluate nanocarrier effects based on the cell shape related parameter circularity to detect cell detachment from the substrate. Albeit this, workflow for data evaluation was feasible and provided insights into cabazitaxel and digitonin toxicity mode of action. Note that in this study, the parameter circularity, which is related to cell rounding, was applied to assess cytotoxicity and was chosen with regard to robustness and effectiveness of data retrieval. However, cell death by apoptosis, necrosis and autophagy can be indicated also by other cell morphological characteristics such as cell swelling or shrinking [31] and the occurrence of cellular debris [22], which can be accessed by DHM. However, therefore, highly robust and reliable automated segmentation of cells in quantitative phase images is required to extract accurately single cell-related parameters as, for example for cells, with close cell–cell contacts growing in clusters, reliable threshold-based image analysis for single-cell data can be highly challenging [72]. Here, sophisticated image segmentation procedures [73] and combination with machine learning algorithms could further increase the quality of the interlaboratory comparison data sets and promise improved extraction of biophysical parameters of individual cells in the future. Machine learning has been applied successfully in earlier studies for the analysis of cell morphology, classification of cells [36] and the identification of cell states SPS:refid::bib74(74). From the images and data presented in Figs. 3A, 4A and 5A, we conclude that automated machine learning-based segmentation and extraction of parameters like motility as well as single cell volume and dry mass could allow a further in-depth evaluation of organic nanocarrier effects on A549 lung epithelial cells. [74]. Earlier, we have demonstrated a robust cell detection and segmentation to be feasible on RAW 264.7 macrophage QPI images that were generated with a similar DHM system as utilized in this study [73]. In this approach, a Mask Region-based Convolutional Neural Network (73) was applied which prospects improved efficiency by reducing demands for manual preinspection of segmented QPI images from time-resolved measurement prior in the extraction of biophysical cell parameters downstream.

Taken together, our interlaboratory comparison study confirms the applied DHM-based cytotoxicity assay for evaluation of organic nanocarriers as transferable to other laboratories by adequately elaborated SOPs. Moreover, the results show that the QPI image data generated at the two participating laboratories allowed a comparable label-free readout of nanocarrier effects on the commonly available A549 lung epithelial cell line.

## Conclusions

In the frame of an interlaboratory comparison study, performed during the EU Horizon 2020 project “Regulatory Science Framework for Nano(bio)material-based Medical Products and Devices (REFINE)”, a DHM-based in vitro cytotoxicity assay for polymeric nanocarriers was performed by two European laboratories. A DHM system and QPI knowledge was established by the technology developing partner BMTZ at SINTEF Industry in Trondheim, Norway. Conversely, nanomaterials for testing were synthesized and characterized at SINTEF and were sent BMTZ. Both partners were able to quantify effects of digitonin as cytotoxicity control, empty and cabazitaxel-loaded PACA nanoparticles on A549 lung epithelial cell morphology via quantitative phase imaging with DHM. Digitonin was confirmed as an appropriate non-particle cytotoxicity control for the DHM QPI assay. While the quantitative analysis of the dry mass increment *DMI* after 12 h performed by the two partner laboratories only showed a significant effect for 128 µg/mL cabazitaxel-loaded PACA nanoparticles at BMTZ, QPI images showed highly reproducible morphological responses of A549 lung epithelial cell circularity to the organic nanocarriers and digitonin. Extended interlaboratory comparison experiments with larger concentration ranges to determine EC_50_ values and multiple analysis time points, retrieved during longer DHM observations, could further improve the achieved standardization and performance of the evaluated QPI toxicity assay. In addition, the results of our study emphasize the importance of standardizing methods for comparability and reproducibility for an improved comparison of nanoparticles effects in in vitro toxicity studies and for a reliable risk assessment for nanomaterials. We conclude that QPI tools for label-free cytotoxicity assessment of nanoparticles are ready for the transfer into common biomedical laboratories, and standardization of these assay methods could advance research in medical nanotechnology and the quantification of cellular responses to polymeric nanocarriers.

## Supplementary Information

Below is the link to the electronic supplementary material.

Supplementary file1 (DOCX 4281 kb)

## Data Availability

Requests for data and materials should be addressed to Björn Kemper (bkemper@uni-muenster.de).
