# Supplementary material for: Interlaboratory evaluation of a digital holographic microscopy–based assay for label-free in vitro cytotoxicity testing of polymeric nanocarriers
Source: Drug Deliv Transl Res. 2022 Jul 8;12(9):2207–24. doi: 10.1007/s13346-022-01207-5 (PMC9263039; doi:10.1007/s13346-022-01207-5)
Supplement: Supplementary file 1 — Supplementary file1 (DOCX 4281 kb) [file 13346_2022_1207_MOESM1_ESM.docx]

# Supplementary Materials

**Supplementary materials Figure S1: Performance comparison of the two DHM systems applied in the interlaboratory study by using a test chart with an included cell phantom (46). (A)** Bright-field microscopy images and **(B)** DHM QPI images of the phase test chart acquired using the DHM system at laboratory 1 (left) and laboratory 2 (right). Phase data were generated as described in materials and method section “Utilized DHM systems and generation of QPI images”. A comparison of the phase signal background was performed by measuring the mean value of the standard deviation measured in 18 randomly selected ROIs (ROI size: 30 µm x 24 µm). Representative ROIs are depicted in the QPI images in (B). The mean phase contrast background was determined to be 0.096 ± 0.024 rad for DHM system at laboratory 1 and 0.073 ± 0.009 rad for the DHM system at laboratory 2. **(C)** Phase distribution along the cross sections depicted in (B). Differences in the curves in (C) can be explained by parasitic interference patterns (evident in (B) for DHM system DHM 1) caused by the coherence properties of the applied laser light. Typical origins of these disturbances are internal reflections within the optical imaging path that can depend on the individual system alignments.

**Supplementary materials Figure S2:** **Representative bright-field images of A549 lung epithelial cells at time point t = 12 h.** Cells incubated with **(A)** medium and cytotoxicity control **(B)** medium and PACA nanoparticles **(C)** medium and PACA cbz nanoparticles.
